# Surviving COVID-19 in Bergamo province: a post-acute outpatient re-evaluation

**DOI:** 10.1017/S0950268821000145

**Published:** 2021-01-19

**Authors:** Serena Venturelli, Simone Vasilij Benatti, Monica Casati, Francesca Binda, Gianluca Zuglian, Gianluca Imeri, Caterina Conti, Ave Maria Biffi, Maria Simonetta Spada, Emi Bondi, Giorgia Camera, Roberta Severgnini, Andrea Giammarresi, Claudia Marinaro, Alessandro Rossini, Pietro Andrea Bonaffini, Giovanni Guerra, Antonio Bellasi, Simonetta Cesa, Marco Rizzi

**Affiliations:** 1 ASST Papa Giovanni XXIII Hospital, Bergamo, Italy; 2 Fondazione IRCSS Ca’ Granda Ospedale Maggiore Policlinico, Milan, Italy

**Keywords:** COVID-19, follow-up, long-COVID, post-acute, symptoms

## Abstract

Bergamo province was badly hit by the coronavirus disease 2019 (COVID-19) epidemic. We organised a public-funded, multidisciplinary follow-up programme for COVID-19 patients discharged from the emergency department or from the inpatient wards of ‘Papa Giovanni XXIII’ Hospital, the largest public hospital in the area. As of 31 July, the first 767 patients had completed the first post-discharge multidisciplinary assessment. Patients entered our programme at a median time of 81 days after discharge. Among them, 51.4% still complained of symptoms, most commonly fatigue and exertional dyspnoea, and 30.5% were still experiencing post-traumatic psychological consequences. Impaired lung diffusion was found in 19%. Seventeen per cent had D-dimer values two times above the threshold for diagnosis of pulmonary embolism (two unexpected and clinically silent pulmonary thrombosis were discovered by investigating striking D-dimer elevation). Survivors of COVID-19 exhibit a complex array of symptoms, whose common underlying pathology, if any, has still to be elucidated: a multidisciplinary approach is fundamental, to address the different problems and to look for effective solutions.

## Introduction

ASST ‘Papa Giovanni XXIII’ is the principal public hospital of the Bergamo province, serving a population of around 1 110 000. This province has been the hardest hit by the initial coronavirus disease 2019 (COVID-19) epidemic wave in Italy, starting from 22 February, with 2346 notified deaths and an estimated +568% increase in all-cause mortality (5058 excess deaths) over the period 20 February–31 March 2020, compared to the average of the same period in the years 2015–2019 [1]. Acute COVID-19 clinical manifestations have been described in detail [2–4], though little published data are available on the post-acute phase, medium-term complications and potential long-lasting harm. Anecdotal evidence is mounting about the so-called ‘long-COVID’: a hard-to-define syndrome, with some patients complaining of symptoms many months following recovery from the acute phase [5–9].

To mobilise health resources, to address the main clinical problems of survivors and to set public health priorities, in the wake of possible epidemic resurgences, a multidisciplinary evaluation of COVID-19 survivors appears of foremost importance [10–12].

At our institution, we organised a public-funded, dedicated outpatient service to follow-up survivors: by the end of September 2020, 1562 persons had completed their first post-discharge assessment; we present the preliminary data as observed in the first 767 patients (2 May through 31 July).

## Methods

A list of all patients discharged from the emergency department (ED) or admitted to the wards of the hospital, with any condition possibly related to severe acute respiratory syndrome-coronavirus-2 (SARS-CoV-2) infection, was obtained from the hospital electronic health records database. We excluded asymptomatic pregnant women admitted for delivery and asymptomatic patients found positive to the molecular test admitted for planned procedures for other conditions.

All patients in the list, if reachable by phone or mail, have been offered to participate in the programme, with the exclusion of paediatric patients (<18 years). Patients still in-hospital were identified but put on a ‘waiting list’ to be recalled for the programme once discharged.

Enrolment into the programme was on a voluntary basis and required a double-negative nasopharyngeal swab for SARS-CoV-2 RNA (positive cases were re-tested regularly until a double-negative swab was obtained).

Cognitively impaired subjects were accompanied by a caregiver, who helped in providing information about the medical history and in recalling the pre-acute episode health status.

We offered a two-step assessment:

- Step 1: nurse assessment, blood tests (including full blood count, liver function tests, renal function tests, D-dimer, coagulation tests, thyroid function tests and thyroid antibodies, glucose, glycated haemoglobin, lactate dehydrogenase, brain natriuretic peptide, C-reactive protein), chest-X-ray, electrocardiogram, full pulmonary function testing with diffusion, psychological evaluation, assessment of rehabilitation needs.
- Step 2 (three days later): infectious diseases consultation and subsequent referral to primary care or to other specialists (mainly respiratory medicine, cardiology, neurology, endocrinology, physical and rehabilitation medicine, haematology) as deemed appropriate.

Several assessment scales were adopted at step 1.

For psychological evaluation, self-report questionnaires were administered to evaluate post-traumatic stress disorder (PTSD), anxiety and depression symptoms and resilience. The following scales were used: Impact of Events Scale - Revised (IES-R) [13–19], Hospital Anxiety and Depression Scale (HADS) [18–21] and Resilience Scale for Adults (RSA) [22, 23].

Since July, a neuropsychological screening test, the Montreal Cognitive Assessment (MoCA) test [19, 24], was introduced to account for the increasing number of patients complaining of cognitive impairment (such as memory and attention deficit). The scale used was based on equivalent scores from 0 to 4, based on adjustment by age and education level of the MoCA scores [25]. In this modified scale, the ‘pathologic’ score was ‘0’.

The results of the questionnaires were always discussed with the patients at the end of the clinical psychological interview.

For assessment of rehabilitation needs the Barthel Index and the Brief Fatigue Inventory scales were used [26–29]. Conditions pre-existing to the acute COVID-19 episode were scored using these two scales, by asking patients to recall their symptoms.

Ethics approval was granted from ASST ‘Papa Giovanni XXIII’ ethical committee. Data were collected using a Microsoft Access database. Written consent was obtained from all participants at enrolment. All patients had access to the follow-up programme regardless of their decision to participate in the study. The regional health system covered all the costs of the service, except for endocrinology, dermatology and rheumatology referrals [30].

## Results

Up to 31 July, 2965 patients met the criteria (946 discharged from the ED and 2019 who were admitted). Six hundred forty-six of them had died (505 before discharge) and 405 declined participation. Of the remaining 1914, 767 had completed the two-step post-discharge assessment by 31 July. None of these patients declined to provide consent for data collection for this observational study.

COVID-19 was confirmed by a positive SARS-CoV-2-RNA PCR in all but 46 cases, among whom 37 had a positive serology (by LIAISON^®^, DiaSorin, Saluggia VC, until 10 July and by ElecSys^®^, Roche, afterwards). The clinical charts of the remaining nine patients were reviewed by a senior ID specialist and judged ‘probable COVID-19’.

Of the 767 enrolled patients, 252 were female (32.9%). The average age was 63 (s.d. 13.6, range 20–92).

The most relevant baseline patients' characteristics and clinical details of the acute phase are summarised in Table 1.

**Table 1.** Baseline patients' characteristics and clinical details of the acute phase

	*n*	%	Sample size
Age mean (s.d.) in years	63 (13.6)		767
Sex (M/F)	515/252	F = 32.9	767
Body mass index			742
<18.5 – underweight	0	0	
18.5–24.9 – normal weight	242	32.6	
25–29.9 – overweight	334	45	
>30 – obese	166	22.4	
Smoking status			767
None	555	72.4	
Active	33	4.3	
Former	179	23.3	
Comorbidities			767
Hypertension	169	21.7	
Coronary disease	73	9.5	
Diabetes	57	7.4	
Atrial fibrillation	36	4.7	
COPD	36	4.7	
Heart failure	34	4.4	
Autoimmune disease	21	2.7	
Solid malignancy	15	2	
Haematologic malignancy	11	1.4	
>1 comorbidity	158	20	
Days from onset to 1st evaluation: median (IQR)	9 (6–12)		625
Admitted patients	678	88.4	767
ICU-treated patients	66	8.6	767
Transferred to other facilities:	332	49	678
Acute care (of whom in ICU)	145 (32)	21.3 (0.05)	
Post-acute care (nursing homes or rehabilitation)	151	22.2	
Post-acute convalescence (e.g. hotels)	28	4.1	
Duration of hospital stay in days: median (IQR):			678
At our institution, not in ICU	10 (6–18)		
At our institution, with ICU admission	30 (19–41)		
Overall^a^ (without ICU admission)	18 (7–40)		
Overall^a^ (with ICU admission)	58 (40–77)		
Oxygen supplementation			749
Room air	159	21.2	
Nasal prongs	213	28.4	
Venturi mask	77	10.3	
Reservoir mask	105	14	
CPAP or NIMV	133	17.8	
Mechanical ventilation	62	8.3	

COPD, chronic obstructive pulmonary disease.

a Overall stay, including hospital stay at other facilities.

Six hundred and sixty-eight persons were hospitalised with 66 (8.6%) of them requiring admission to the intensive care unit (ICU).

Eight per cent of the admitted patients had a total hospital stay of more than 60 days.

Survivors entered our programme at a median of 81 days (IQR = 66–106) after discharge from the ED or from the wards, and a median of 105 days (IQR = 84–127) after the appearance of the first symptoms related to COVID-19.

On the day of the first visit at our service, they had been at their home for a median of 68 days (IQR = 51–92) (332 patients, 43% of our cohort, was transferred to other facilities before returning home).

The main results are summarised in Table 2.

**Table 2.** Follow-up multidisciplinary assessment

	*n*		%		Sample size	
Patients feeling recovered	510		66.5		767	
Days since patients have felt recovered, median (IQR)	49 (30–72)				510	
Persistent symptoms at follow-up visit						
	F	M	F	M	F	M
None	97	276	38.5	53.6	252	515
Confusion	7	16	2.8	3.1	252	515
Asthenia	93	93	36.9	18.1	252	515
Dyspnoea	66	101	26.2	19.6	252	515
Fever	1	3	0.4	0.6	252	515
Myalgia	9	20	3.6	3.9	252	515
Cough	8	15	3.2	2.9	252	515
Headache	1	3	0.4	0.6	252	515
Chest pain	9	15	3.6	2.9	252	515
Palpitations	18	12	7.1	2.3	252	515
Syncope	0	1	0.0	0.2	252	515
Anosmia/Dysgeusia	11	12	4.4	2.3	252	515
Upper gastro-intestinal symptoms	1	0	0.4	0.0	252	515
Lower gastro-intestinal symptoms	4	3	1.6	0.6	252	515
Others^a^	13	46	5.2	8.9	252	515
⩾3 persistent symptoms	7	0	2.8	0.0	252	515
Oxygen saturation on room air						
Mean (range)	97.8 (91–100)				767	
⩾95%	722		94.1		767	
93-94%	44		5.7		767	
<93%	1		0.1		767	
Modified Medical Research Council dyspnoea Scale (mMRC)						
Grade 0 – No dyspnoea	539		70.3		767	
Grade 1 – Mild dyspnoea	176		23.0		767	
Grade 2 – Moderate dyspnoea	42		5.5		767	
Grade 3 – Severe dyspnoea	10		1.3		767	
Grade 4 – Very severe dyspnoea	0		0.0		767	
Karnofsky performance status scale						
>90%	640		90.9		704	
80-90%	51		7.2		704	
<80%	13		1.8		704	

	*n*	%	Sample size
***Physical medicine evaluation***			
Barthel Index prior to COVID-19			
Total independent	690	91.0	758
Semi-independent	34	4.5	758
Slight dependency	14	1.8	758
Moderate dependency	11	1.5	758
Severe dependency	6	0.8	758
Total dependency	2	0.3	758
Barthel Index at follow-up			
Total independent	569	75.1	758
Semi-independent	138	18.2	758
Slight dependency	20	2.6	758
Moderate dependency	16	2.1	758
Severe dependency	11	1.5	758
Total dependency	3	0.4	758
Brief Fatigue Inventory prior to COVID-19			
No asthenia	553	73.0	758
Mild asthenia	134	17.7	758
Moderate asthenia	53	7.0	758
Severe asthenia	18	2.4	758
Brief Fatigue Inventory at follow-up			
No asthenia	219	28.9	758
Mild asthenia	98	12.9	758
Moderate asthenia	247	32.6	758
Severe asthenia	194	25.6	758
***Psychological evaluation***			
IES-R scale			
Normal	505	69.5	727
Pathologic	222	30.5	727
HADS-Anxiety			
Normal	645	88.7	727
Pathologic	82	11.3	727
HADS-Depression			
Normal	694	95.5	727
Pathologic	33	4.5	727
RSA			
Normal	679	95.5	711
Pathologic	32	4.5	711
MoCa			
Normal	302	99.3	304
Pathologic	2	0.7	304

	*n*	%	Sample size
**Pulmonary Function Tests with CO diffusion^a^**			
FEV1 (l), median (IQR)	2.97 (2,38–3.7)		716
FEV1% of expected, median (IQR)	98 (87–109)		716
FVC (l), median (IQR)	3.75 (3–4.55)		717
FVC% of expected, median (IQR)	95 (84–106)		717
DLCO (ml/min/mmHg), median (IQR)	23.43 (18.16–29.16)		680
DLCO% of expected, median (IQR)	96 (81–112)		680
FEV1/FVC, median (IQR)	0.81 (0.76–0.85)		716
FEV1/FVC LLN, median (IQR)	0.65 (0.63–0.68)		716
Obstruction prevalence	3.7%		
Restrictive pattern prevalence	11.8%		
Mixed pattern prevalence	0.9%		
DLCO reduction	19%		
**Blood tests: principal findings**			
D-Dimer (ng/ml), mean (s.d.)	700 (1021)		743
D-Dimer 500–999	163	22	743
D-Dimer 1000–1999	88	12	743
D-dimer ⩾2000	39	5	743
LDH (U/l), mean (s.d.)	222 (42)		756
LDH >256	124	16	756
Lymphocytes (cells/μl)			
Lymphocytes <3400	707	93	761
Lymphocytes <1000	28	4	761
C-reactive protein (mg/dl), mean (s.d.)	0.36 (0.85)		
C-reactive protein >1.0	52	7	759
Creatinine (mg/dl)			
Creatinine >1.1 (females)	17	7	249
Creatinine >1.3 (males)	37	7	509
TSH (μU/ml)			
TSH >5 OR <0.3	24	4	657
Anti-thyroid antibodies (UI/ml)			
Anti-Thyroglobulin Ab >60	61	9	656
Thyroid peroxidase Ab >60	115	18	656

**Complications**	F	M	F	M	F	M
None	176	338	69.8	65.6	252	515
Pulmonary (bacterial pneumonia, pleural effusion, pneumothorax)	20	35	7.9	6.8	252	515
Cardiac – arrhythmia	10	29	4.0	5.6	252	515
Cardiac – ischaemia	2	12	0.8	2.3	252	515
Cardiac – inflammatory	2	10	0.8	1.9	252	515
Renal (acute renal failure)	5	11	2.0	2.1	252	515
Thrombotic (pulmonary embolism, deep-vein thrombosis)	10	37	4.0	7.2	252	515
Haemorrhagic	3	6	1.2	1.2	252	515
Psychiatric (delirium, depressive syndrome, psychosis)	5	12	2.0	2.3	252	515
Neurologic (CNS: stroke, encephalitis)	6	20	2.0	3.5	252	515
Neurologic (PNS: Guillain Barré syndrome, polyneuropathy)	6	20	2.4	3.9	252	515
Infective (excluding pneumonia)	7	18	2.8	3.5	252	515
Rheumatologic/Dermatologic	11	11	4.4	2.1	252	515

**Referrals**			
No referral (end of follow-up)	388	50.6	767
Pulmonary medicine consultation	281	36.6	767
	*n*	%	Sample size
Cardiology consultation	63	8.2	767
Neurology consultation	52	6.8	767
Endocrinology consultation	41	5.3	767
Haemostasis and thrombosis Centre consultation	40	5.2	767
Diabetology consultation	20	2.6	767
Others^b^	7	0.9	767

COPD, chronic obstructive pulmonary disease; IES-R, Impact of Event Scale-Revised; HADS, Hospital Anxiety and Depression Scale; RSA, Resilience Scale for Adults; MoCA, Montreal Cognitive Assessment; LDH, lactate de-hydrogenase; TSH, thyroid stimulating hormone; CNS, central nervous system; PNS, peripheral nervous system.

a FEV1: forced expiratory volume in the 1st second; FVC: forced expiratory volume); LLN: lower limit of normality; obstruction is defined by FEV1/FVC values below the LLN; restrictive pattern is defined by FVC values below the LLN; mixed pattern is defined by coexistence of obstruction and restrictive pattern; DLCO is considered reduced when below the LLN. All reference values are calculated with GLI 2012 for spirometry and GLI 2017 for DLCO equations.

b Nephrology consultation, gastroenterology consultation, vascular surgery consultation.

At the time of ID evaluation, 394 patients (51.4%) reported being still symptomatic, with fatigue and exertional dyspnoea as the most reported symptoms. Women were more symptomatic than men, with fatigue reported almost twice as frequently. Out of the symptomatic patients, 257 (33.5%) stated they were ‘not feeling fully recovered’, when specifically asked. The remaining 137 patients (17.9%) experienced only minor symptoms with little or no implications for their daily activities: as such, they considered themselves ‘recovered’.

One hundred and eighty-six patients (24.2%) were still on additional medical treatment, introduced during admission, with anticoagulants being the most frequent.

Self-reported dyspnoea using the Modified Medical Research Council Dyspnoea Scale (mMRC) was present in 228 patients (29.8%), of whom 52 patients had ‘moderate−severe’ dyspnoea.

Based on Barthel index scale results 121 patients (16%) were no longer fully independent, of these only six became moderately−severely dependent. The Brief Fatigue Inventory test found 334 patients (44.1%) complaining of new-onset fatigue (145 with moderate−severe fatigue).

While the Impact of Event Scale-Revised (IES-R) identified 222 patients (30.5% of 727) with COVID-19-related traumatic aspects, the RSA highlighted for 679 (95.5% of 711) they had enough resources to react. The MoCa screening, introduced in July, was pathologic in just 2 out of the 304 patients who were tested, despite 69 reporting related symptoms. Pulmonary function testing identified 27 patients (3.7%) with obstruction, 85 (11.8%) with restrictive pattern and 6 (0.9%) with mixed pattern. For 51 patients the test result was contraindicated or not diagnostic. Diffuse capacity of the lungs for carbon monoxide (DLCO) was reduced in 19% of patients.

C-reactive protein, D-dimer and lactate dehydrogenase were above the upper limit of normal (ULN) in 7%, 38% and 22% of cases, respectively. Two asymptomatic pulmonary sub-segmental thrombosis were discovered at follow-up, by investigating striking D-dimer elevation.

Anti-thyroglobulin and thyroid peroxidase antibodies were found elevated in 15% of the patients, with 5% of them (6 out of 115) showing concomitant derangement of the thyroid-stimulating hormone (TSH).

Two hundred and fifty-three patients (32.9%) had SARS-CoV-2 related complications during the acute phase, of whom the most frequent ones were:

- Neuropsychiatric (8.7%) (e.g. delirium, depressive syndrome, psychosis, stroke, encephalitis, Guillain−Barré syndrome, polyneuropathy).
- Cardiac (8.5%) (e.g. arrhythmia, ischaemia, myocarditis)
- Pulmonary additional complications (7.1%) (e.g. bacterial pneumonia, pleural effusion, pneumothorax).
- Thrombotic (6.1%) (e.g. pulmonary embolism, deep-vein thrombosis).

Complication almost exclusively occurred during the acute phase, but were assessed at follow-up to avoid missing the ones of late appearance.

Following ID evaluation, 379 patients (49.4%) were referred to specialty pathways. The majority (281 patients; 36.6%) were referred to respiratory medicine. Several patients were referred to more than one specialty.

## Discussion

To our knowledge, our report represents one of the largest cohort to date, describing the medium-term consequences of SARS-CoV-2 infection.

It is a mono-centric study, referring to a homogeneous population, with a large majority (88.4%) of patients admitted to hospital of which 9.7% requiring intensive care: in other published cohorts so far, the selection criteria were based on just having a positive molecular test [31, 32].

Of the admitted patients in our cohort, 43% were transferred to other facilities at least once during the hospital stay, experiencing what could be described as a ‘journey’ between different hospitals and hospital services (8% of patients had a total hospital stay of more than 60 days). We tracked down all the accessible information, for each case, along all these movements.

Another strength of this study is that patients were actively searched for (by phone and mail) and physically examined and interviewed (in some cases, with the help of a caregiver): this provided equal access to the follow-up service, overcoming barriers posed by telephone and video consultations. Lastly, as long as COVID-19 appears to affect different organ systems, in some cases even without any relevant pulmonary disease, our large criteria of selection allowed us to depict a wide scenario of the consequences of COVID-19 in our population.

Patients surviving COVID-19 show a complex array of conditions, ranging from mild to potentially life-threatening (e.g. late-onset pulmonary embolism). Our intervention had the advantage of assessing health needs and, at the same time, offering interventions to address them, within the WHO-endorsed framework of ‘Recognition, research and rehabilitation’, as invoked for long-COVID sufferers [33].

Three hundred and ninety-four participants (51.4%) were still symptomatic at the ID evaluation, which is in line with other authors' observations [31, 32, 34, 35]. In particular, 33.5% described themselves as ‘not yet recovered’: at a median time of 105 days since onset, these people probably fit into the ‘working definition’ (still to be strictly defined) of ‘long COVID sufferers’[8].

The nature of the long-COVID symptoms has yet to be explained, however it appears reasonable to try and separate those symptoms related to post-viral chronic fatigue syndrome, from those due to post-critical-illness syndrome, or post-traumatic stress disorder, especially in a cohort with a high rate of hospitalisation and a long length of stay, as ours.

In this effort, a comprehensive and multidisciplinary assessment of patients after the acute episode of COVID-19 should be considered.

We adopted for each patient various scales of self-evaluation, comprehensive laboratory and instrumental tests: their correlation to the reported symptoms and their cross-concordance will be object of our next efforts.

Mid-term consequences of COVID-19 are not limited to lung disease and cover a wide range of organ systems: the underlining mechanism could be some kind of microcirculatory impairment [36, 37]. We propose that studies should be addressed in this direction.

Our study has a number of limitations. Firstly, the timeline of enrolment and assessments was not standardised: we have seen patients at variable intervals from the onset of COVID-19, making inter-group comparisons less stringent; this has to do with the immediate establishment of our intervention, right after the end of the first wave of the epidemic. Secondly, our inclusion criteria are pragmatic, but hardly representative of the actual case load in Bergamo province; during the initial epidemic, confounding factors altered the hospital case mix in both ways: severely ill patients did not gain access, due to ambulance system breakdown and less severely affected patients avoided consulting the ED due to overcrowding. Thirdly, the criterion of a double-negative test before enrolment delayed the evaluation of a part of our cohort: it's not yet known if patients having a persistent positive swab share any relevant clinical characteristics, so that this delay could act as a source of variability. Fourthly, recall bias could have affected the scores given to the ‘pre-COVID’ assessment. Lastly, we censored our observations at the ID consultation, with a relevant percentage of the enrolled patients still to be seen by other specialists, who, in turn, may add further perspectives.

In conclusion, a large proportion of survivors of COVID-19 from our setting had significant ongoing health and psychosocial needs. Provision of a coordinated, multidisciplinary follow-up clinic offering a comprehensive medical and psychological assessment should be considered for such patients. Further research is required to better understand the burden of morbidity after acute COVID-19 infection, in order to plan and fund appropriate services.

## Data Availability

The data that support the findings of this study are available on request from the corresponding author (sventurelli@asst-pg23.it). The data are not publicly available due to their containing information that could compromise the privacy of research participants.
